# Associated systemic diseases and etiologies of medication-related osteonecrosis of the jaw: a retrospective study of 265 surgical cases

**DOI:** 10.1186/s40902-023-00377-7

**Published:** 2023-02-28

**Authors:** Hoon-Je Chang, Min-Jae Kim, Kang-Min Ahn

**Affiliations:** Department of Oral and Maxillofacial Surgery, Asan Medical Center, College of Medicine, University of Ulsan, Seoul, South Korea

**Keywords:** Osteonecrosis of the jaw, Osteoporosis, Metastatic cancer, Bisphosphonate, Denosumab, Bone targeting agents

## Abstract

**Background:**

Medication-related osteonecrosis of the jaw (MRONJ) is one of the complications caused by various drugs. As there are increasing reports of MRONJ, it is important to diagnose and identify patients who have the potential risk of the disease. The aim of this study was to analyze the systemic diseases, etiology, and treatment results of MRONJ.

**Material and methods:**

A total of 265 MRONJ operations were reviewed retrospectively. This study included patients who were diagnosed as MRONJ and those who also received surgery, ranging from simple extraction to reconstruction with free flaps, from 2009 to 2021. Each patient’s systemic disease and eitology and basic demographic information was taken into consideration.

**Results:**

The most common diseases related were osteoporosis (*n* = 127), breast cancer (*n* = 77), multiple myeloma (*n* = 27), prostate cancer (*n* = 26), and etc. (*n* = 12). The related causes of MRONJ were extraction (*n* = 138), implants (*n* = 40), and irritations by prosthesis (*n* = 29); however, 55 cases were occurred spontaneously. Out of 265 patients, 214 were women while 51 were men. The average age when the surgery took place was 67.7 and 69.8 years for male and female patients, respectively. Saucerization and sequestrectomy (*n* = 252) was the most common surgical treatment, followed by mandibulectomy (*n* = 12) and partial maxillectomy (*n* = 1). While 4 cases occurred in both jaws, 168 cases were in the mandible and 93 cases were in the maxilla.

**Conclusion:**

Nearly 50 % of the MRONJ patients had osteoporosis and the other patients who received bone targeting agents parentral had bone metastasis of various cancers. Extraction is the most common related event for MRONJ.

## Background

Medication-related osteonecrosis of the jaw (MRONJ) is one of the complications caused by bisphosphonates and antiresorptive drugs. It is defined as the exposure of bone or bone that can be probed through an intraoral or extraoral fistula in the maxillofacial region that has persisted for more than 8 weeks and has no history of radiation therapy to the jaws or metastatic disease to the jaws [1]. As reports of MRONJ are gradually increasing, the need for dental practitioners to diagnose and evaluate the patients who have potential risk of the disease is getting more important [2]. Even though the prevalence of MRONJ is relatively low, there may be patients or dental practitioners that are unaware of the disease.

Bisphosphonates are effective in preventing bone fractures. Used as the correct amount, it maintains the bone’s mechanical properties and this is used as a typical treatment in osteoporosis patients. The nitrogen-containing bisphosphonates interfer the mevalonate pathway and inhibits protein prenylation impairing osteoclast formation and causing osteoclast apoptosis [3]. Denosumab, a receptor activator of nuclear factor kappa-B ligand (RANKL) monoclonal antibody, binds to receptor activator of nuclear factor kappa-B (RANK) inhibiting RANKL to bind with osteoclast precursors. Impaired precursor differentiation results in reduced osteoclast formation and function, decreasing bone resorption [4]. For cancer patients, for example, breast cancer and prostate cancer, bisphosphonates and denosumab are used frequently to reduce metastasis to the bone. These antiresorptive agents are also used to reduce skeletal-related events in multiple myeloma patients [5]. However, the overall bone remodeling cycle is impaired by direct or indirect reasons of antiresorptive agents, resulting in necrotic bone formation.

The importance of identifying risk factors in patients who are susceptible to developing MRONJ is increasing. A study which is conducted under a consistent protocal of surgical indications is necessary. So far, there is no report of a review on a large amount of patients that underwent surgery by one surgeon in one institution in South Korea. Scrupulous medical history taking and selective dental procedures are emphasized to be aware of MRONJ. The pupose of this study was to discriminate the possible risk factors and the related diseases of MRONJ.

## Materials and methods

Patients who received surgery from 2009 to 2021 due to MRONJ was reviewed. The study was approved by the institutional review board (S2022-2366-0001). The surgery included from simple sequestrectomy and saucerization to reconstruction with free flaps that were performed by a single surgeon at the department of oral and maxillofacial surgery. A total of 265 operations were analyzed by the etiological factors such as the systemic disease and the risk factor or adjunctive procedures that causes MRONJ. The research including factors like the age, sex, trigger factor, and systemic disease was performed by two investigators. The inclusion criteria were patients who were diagnosed as any MRONJ stage, according to the American Association of Oral and Maxillofacial Surgeons (AAOMS) guidelines and underwent surgery due to the identical disease. Patients who had a history of radiation therapy at the jaws were excluded. The patient was examined by clinical evaluation, panoramic views, and medical data. The patient’s age, sex, medication history, and systemic disease were recorded.

The surgical operation was performed by the following criteria: (1) sequestrum formation when it is radiologically reviewed or when the movement of the sequestrum is certain clinically examined, (2) when the patient is suffering from pain despite the antibiotic and pain killing drug therapy is used, and (3) patients who are capable of receiving operations and are suffering from MRONJ stage 3 which includes those who have exposed necrotic bone beyond the alveolar bone, pathologic fracture, extraoral fistula, oroantral or oronasal communication, osteolysis near the mandible margin or sinus floor according to AAOMS. Prior to surgical treatment, precise consultation to the patient’s physician was sent to arrange the schedule for operation in case the patient was going under chemotherapy.

## Result

**Fig. 1:**
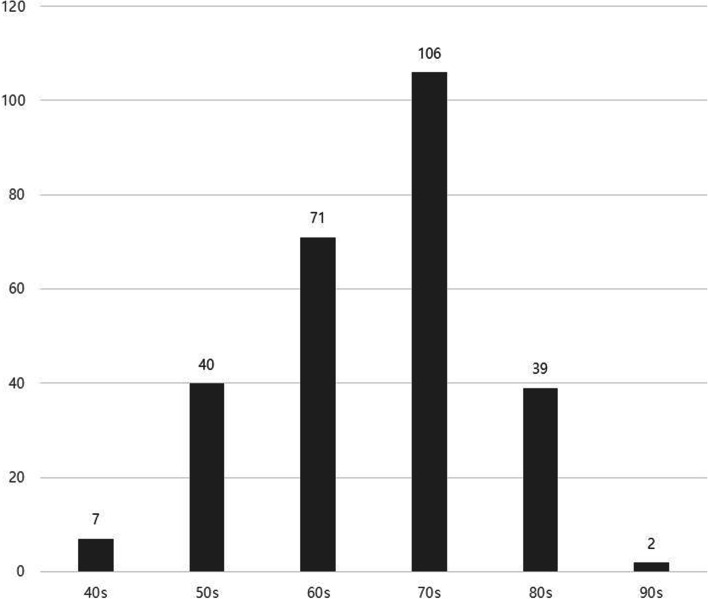
Distribution of the age by decades in medication-related osteonecrosis of the jaw

A total of 265 patients (W: 214 vs M: 51) underwent surgical operation. The mean age was 69.8 ± 11.1 and 67.7 ± 6.5 years, respectively, at the moment the patients underwent surgery and the overall mean age was 69.3 years. Distribution of patients’ ages by decades are depicted in Fig. 1.

The most common diseases related with MRONJ were osteoporosis (*n* = 127), breast cancer (*n* = 77), multiple myeloma (*n* = 27), prostate cancer (*n* = 26), and etc. (*n* = 12). There were two patients who had osteoporosis and breast cancer together and two patients who had osteoporosis and renal cell cancer together. Out of 127 patients who had osteoporosis, only 6 patients were male, and medication change to teriparatide took place in 10 cases. A total of 6 patients were receiving treatment due to renal cell cancer, male and female patients in equal numbers. Two patients, one male and one female, were on medication of imatinib (Glivac). Four patients received an operation because of MRONJ, but the medical history was not taken sufficiently. The classification of diseases by gender is shown in Table 1. Also, out of the 265 cases of MRONJ, 67 patients had diabetes mellitus and 84 patients were receiving steroid treatment.

**Table 1:** Systemic diseases related to medication-retated osteonecrosis of the jaw

Systemic disease	Male	Female
Osteoporosis	6	121
Multiple myeloma	15	12
Breast cancer	0	77
Prostate cancer	26	0
Renal cell cancer	3	3
Gastrointestinal stromal tumor	1	1
Unknown	0	4

According to the AAOMS staging system, only 1 patient was MRONJ stage 0 and a total of 23, 199, and 42 patients were classified as MRONJ stage 1, 2, and 3 respectively. The mandible (*n* = 168) was the more common site of MRONJ occurence than the maxilla (*n* = 93) and there were four cases which occurred in both jaws.

The suggested causes of MRONJ were extraction (*n* = 138), implants (*n* = 40), irritations by denture (*n* = 29), and irritations by bridge pontics (*n* = 3); however, there were 55 cased of spontaneous occurrence. There was no difference in the most frequent trigger point for MRONJ in men and women. The classification of diseases by gender is shown in Table 2.

**Table 2:** Risk factors or adjunctive procedures associated with medication-retated osteonecrosis of the jaw

Risk factor or adjunctive procedures	Male	Female
Extraction	25	113
Spontaneous occurance	14	41
Implants	9	31
Irritations by denture	3	26
Irritations by bridge pontics	0	3

The most frequently perfomed surgery was saucerization and sequestrectomy (*n* = 252) and mandibulectomy (*n* = 12) and partial maxillectomy (*n* = 1) were the following treatments. Out of nine mandibulectomy patients, four received fibular free flap reconstruction and all were successful.

In addition, four representative cases of MRONJ surgical treatment that is regulary taking place in our institute are demonstrated. Of each medical disease that is related to MRONJ, nonsurgical treatment was considered preferentially, and surgical intervention was planned by the patient’s symptom and overall situation. We consider that these cases could give information to surgeons regarding the moment of surgical intervention.

**Fig. 2:**
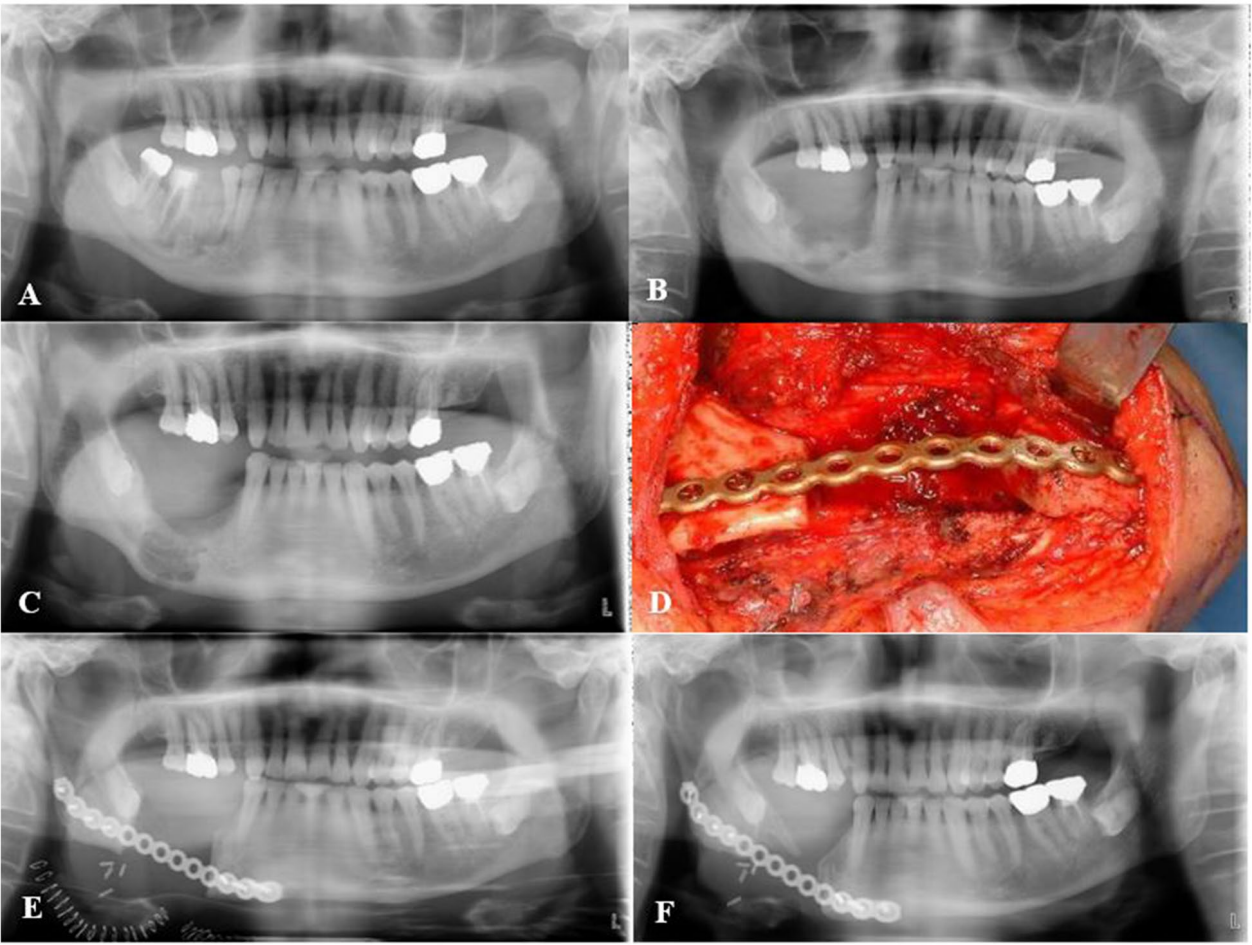
Surgical treatment of medication-related osteonecrosis of the jaw in prostate cancer patient. **A** Preoperative panoramic radiograph showing osteonecrosis of the right mandible in #45 area. **B** Postoperative panoramic radiograph after 2 weeks. **C** Postoperative panoramic radiograph after 1year showing the aggravation of the disease. **D** Partial mandibulectomy and reconstruction plate application. **E** Postoperative panoramic radiograph after 1day of mandibulectomy. **F** Postoperative panoramic radiograph after 1 year of mandibulectomy showing stable results

One patient who was receiving treatment of prostate cancer visited the oral and maxillofacial surgery department due to pain in the right mandibular area. The patient had a medication history of 14 times of zoledronate 4 mg injection for 1 year. Although the symptoms were reduced when the antibiotic treatment was initiated, the bone was still in exposed state and later a fistula was formed at the buccal mucosa. The patient underwent saucerization and extraction and seemed to heal well. However, at a 1 year postoperative state, the disease was aggravated and the lesion was extended to the mandible border. As a result, partial mandibulectomy was performed and the postoperative results were stable (Fig. 2).

**Fig. 3:**
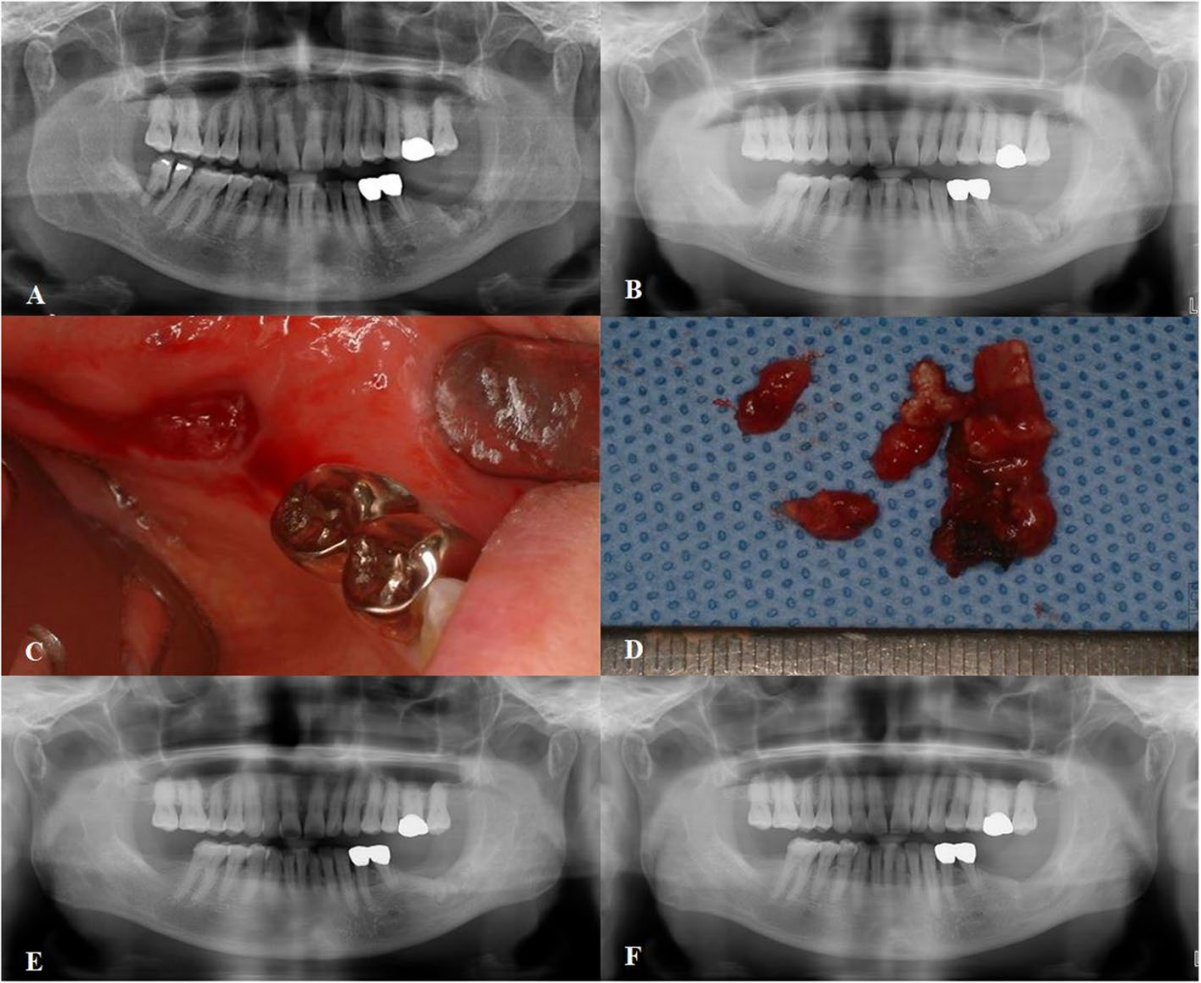
Surgical treatment of medication-related osteonecrosis of the jaw in osteoporosis patient. **A** Preoperative panoramic radiograph showing sequestrum formation in #37 extraction socket. **B** Panoramic radiograph of 3 months after antibiotic treatment. **C** Inflammation at #37 extraction site. **D** Removed necrotic bone after saucerization and sequestrectomy. **E** Panoramic radiograph of 3 years after antibiotic treatment showing the remaining sequestrum in #37 extraction socket. **F** Postoperative panoramic radiograph after 2 weeks

A patient was refered to oral and maxillofacial surgery for unsufficient healing after extraction. The patient was undergoing medication treatment for osteoporosis. Antibiotic treatment was intially used to relieve the symptoms. After 3 years, the patient revisited our department due to the pain in the left mandibular area. Since sequestrum formation was certain in radiologic views, saucerization was taken in place and the postoperative results were stable as anticipated (Fig. 3).

**Fig. 4:**
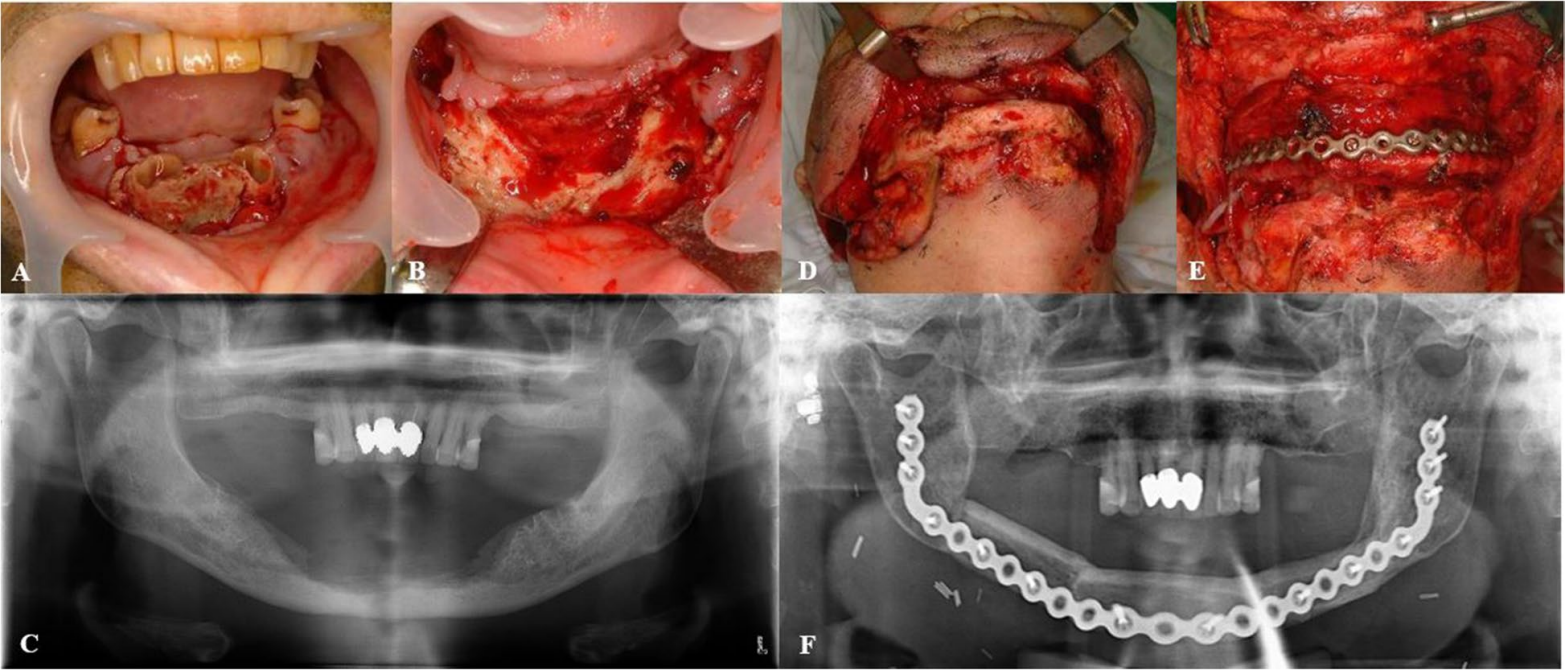
Surgical treatment of medication-related osteonecrosis of the jaw in multiple myeloma patient. **A** Preoperative state showing exposed bones anterior mandible. **B** Saucerization and extraction performed. **C** Postoperative panoramic radiograph after 1day of saucerization. **D** Progressed disease in the mandible. **E** Reconstruction of the mandible with fibular free flap and reconstruction plate. **F** Postoperative panoramic radiograph after 2 years showing stable results

Another case was a patient who had multiple myeloma as underlying disease. The patient was undergoing medication treatment with pamidronate 90 mg injection. The patient was suffering from exposed bone and numbness of the mandible, so surgery was perfomed after medical consultation to the department of hematology. Saucerization of the mandible and extraction of the left third molar and both first and second molars were done and the patient’s symptoms were temporarily improved. However, after 6 months from the initial surgery, the bone was reexposed and denudation was proceeding. Since infection was hard to control and there was a large possibility of pathologic fracture, partial mandibulectomy and fibular free flap reconstruction was planned. The surgery was successful and the patient showed good postoperative results (Fig. 4).

**Fig. 5:**
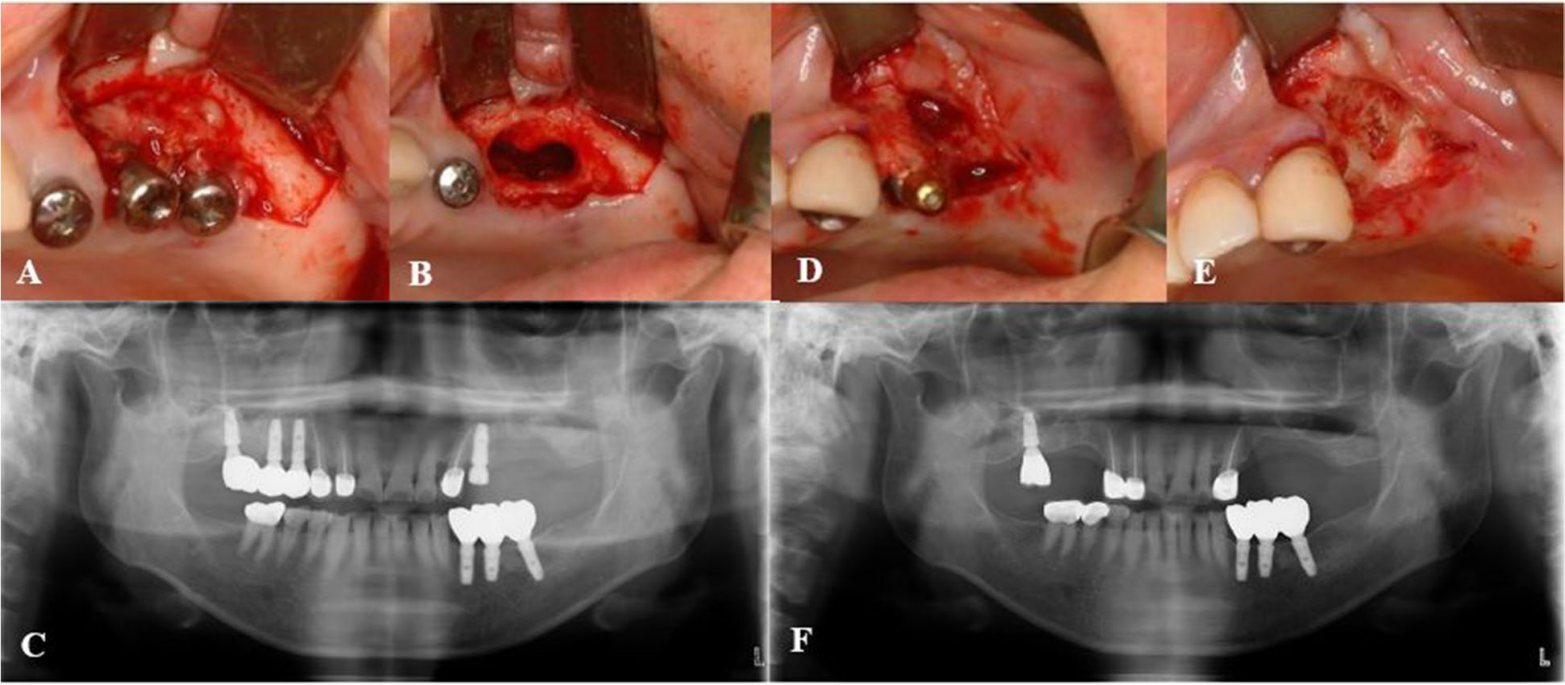
Surgical treatment of medication-related osteonecrosis of the jaw in breast cancer patient. **A** Inflammation at #26, 27 implant area. **B** Implant removal and saucerization at #26, 27 area. **C** Postoperative panoramic radiograph after 3 days showing clean resection of the lesion. **D** Inflammation at #24 implant area after 1 year and 2 months after the initial surgery. **E** Implant removal and saucerization performed. **F** Postoperative panoramic radiograph after 5 years and 3 months showing stable results at both surgical sites

One patient with breast cancer and bone metastasis visited our department because of inflammation and swelling on her left maxillary area where she received implant treatment. Bone exposure was examined and the patient was suffering from inflammation and swelling. Implant removal and saucerization was performed along with antibiotic treatment. However, sequestrum in her right maxillary area where implant treatment was done was also examined. Additional implant removal and saucerization was performed after 3 months since the first surgery. After 1 year, pus discharge was examined from the left first premolar implant area. Eventually, the implant was removed and saucerization was done. The patient showed excellent healing state and the operation results remained stable for over 5 years (Fig. 5).

## Discussion

The etiology of MRONJ is still not fully understood, besides the fact that it has relation with the slow turnover of bone due to antiresorptive medication and a trigger point such as inflammation. There are hypotheses that are rather specific, including bone remodeling inhibition, inflammation or infection, angiogenesis inhibition, innate or acquired immune dysfunction, and genetic factors, and it is getting more evident that the induction of MRONJ is of several causes rather than one [1]. This study was carried out to figure out which systemic disease has the most relation with the prevalence of MRONJ. The study’s significance comes from the large number of patients and data that was analyzed retrospectively.

A total of 265 patients were included and 214 were female while 51 were male. The condition from which underlying disease the medication is prescribed is probably the reason for the higher prevalence of MRONJ in women. Postmenopausal osteoporosis is a type of osteoporosis that is common that affects many women. Antiresorptive agents (bisphosphonates and denosumab) are frequently used to effectively reduce bone resorption [6]. Although the prevalence of MRONJ is higher in metastatic breast cancer than in osteoporotic patients, the relatively higher portion in osteoporosis patients is likely because of the difference in the total number of patients [1, 7].

The most common direct cause that induced MRONJ was extraction followed by spontaneous occurrence. Spontaneous occurrence is a factor that is frequently related to MRONJ. It may be the result of not taking the patient's medical history thoroughly or the patient’s unawareness of the medication therapy he or she had received. However, it is a common factor besides extraction and further investigation related with the pathophysiology seems required [8, 9]. As in this study, MRONJ is usually a complication which patients treated with breast cancer, prostate cancer, multiple myeloma, and prostate cancer have. However, there are two patients who developed MRONJ due to imatinib in this study, and several reports currently show that imatinib is also a drug that has a relation [10–12].

As recommended in the AAOMS 2022 position paper, changing the medication from antiresorptive agents to teriparatide in osteoporosis partients is effective and many other studies support these results [1, 13, 14]. However, only 10 of the patients were confirmed of treatment change to teriparatide in the result. Only the patients who are receiving treatment in the identical medical institute could be meticulously followed. A large proportion of patients receive their medication from local clinics making it difficult to check for medication change. Consultation with the patient’s physician is necessary, and intially, calcium and vitamin D supplements are used usually instead.

In our result, a large proportion of the patients had diabetes mellitus (25.3%) and were going under steroid treatment (31.7%). Studies that indicate diabetes mellitus and steroid treatment as risk factors are continuously being published. Although the evidence that diabetes mellitus is a causation of MRONJ is unsufficient, researches seem to show that diabetes mellitus and MRONJ have a strong association [15, 16]. Diabetes mellitus impedes oral wound healing by increased inflammation and infection possibilities and microvascular damage, and many other pathogenesis are described but the direct mechanism is still not certain [17, 18]. Steroids are widely used in cancer patients, but are also known for risk factors of osteonecrosis, including MRONJ. Steroid treatments can have association with MRONJ due to their potential role in apoptosis, endothelial cell damage, and coagulation pathway. It is known that those who are older and received longer drug use period or higher dose usage have higher possibilites of osteonecrosis [19].

Nonoperative therapies should be considered preferentially because it can be selected at any MRONJ stage. Surgical intervention was implemented when the presented criteria were met. According to the AAOMS position paper-2022 update, operative therapies should be approached as a treatment option to reduce MRONJ because it has an unpredictable character and does not always have optimal results [1]. Still, many studies have shown successful treatments of MRONJ by removing necrotic bones by surgical approaches [20–22]. It is important to classify the resection margin according to the stage of MRONJ, but the most important thing is that the resection margin should be set until the vital bone, when bleeding appears beyond the necrotic bone margin. Several studies suggest the benefits for early operations, suggesting an early approach in surgical intervention [20, 23].

However, although early surgical intervention can improve the disease, a more apparent criteria to determine the exact period is required. First, surgical intervention should be performed when primary closure is possible. The need to achieve mucosal integrity rapidly can not only be a reason for early operations but also signifies that when primary closure is possible, surgical procedures can take place [24]. In the case of maxilla surgeries, the buccal fat graft can be used to acquire primary healing [25, 26]. Second, when mucosal integrity can be achieved. Soft tissue healing in MRONJ patients are disturbed due to the medication effects of antiresorptive agents. Angiogenesis inhibition and the impairment of wound differentiation can be the reasons for insufficient soft tissue healing [27, 28]. Since the soft tissue cannot be covered over the necrotic bone tissue, when a sequestrum shows enough mobility, removing the sequestrum by an operative therapy can lead to mucosal integrity and this can shorten the treatment period. Third, when it is evaluated as the third stage of MRONJ. In this case, the patient is experiencing clinical symptoms and pain and is in a situation of extreme discomfort due to pathologic fracture of extraoral fistula. If the patient’s general condition is good, immediate surgical intervention is necessary because the benefits that are obtained from surgery are greater than the risks of not receiving operative therapies. Usually after the surgery, the patients can enjoy a much comfortable life since the pain, swelling, and discomfort is decreased instantly [29].

The limitation of this study is that it is based on the medical records which were not precisely designed for the study. As this study is retrospective, some medical records may not have been meticulously written down. Therefore, the data collected made it difficult to identify some precipitating events of MRONJ. Also, several patients’ follow-up appointments were unregular and even lost, making it unable for further investigation.

## Conclusions

MRONJ is a severe complication in patients who received bisphosphonates and denosumab. Nearly 50% of the MRONJ patients had osteoporosis and the other diseases are bone metastasis of various cancers. Surgeons must be aware of patients receiving drug therapy and must take medical records precisely to identify the related systemic disease and risk factor. Since minor oral procedures which could expose the alveolar bone such as extraction and implant surgery can induce MRONJ, patients who have received treatments with bone-targeting agents should be treated by an oral and maxillofacial surgeon.

## Data Availability

Not applicable

## Abbreviations

AAOMS: American Association of Oral and Maxillofacial Surgeons
MRONJ: Medication-related osteonecrosis of the jaw
RANK: Receptor activator of nuclear factor kappa-B
RANKL: Receptor activator of nuclear factor kappa-B ligand
